# High Content Screening as High Quality Assay for Biological Evaluation of Photosensitizers In Vitro

**DOI:** 10.1371/journal.pone.0070653

**Published:** 2013-07-29

**Authors:** Gisela M. F. Vaz, Edyta Paszko, Anthony M. Davies, Mathias O. Senge

**Affiliations:** 1 Medicinal Chemistry, Institute of Molecular Medicine, Trinity Centre for Health Sciences, Trinity College Dublin, St. James's Hospital, Dublin, Ireland; 2 Department of Clinical Medicine, Institute of Molecular Medicine, Trinity Centre for Health Sciences, Trinity College Dublin, St. James's Hospital, Dublin, Ireland; 3 School of Chemistry, SFI Tetrapyrrole Laboratory, Trinity Biomedical Sciences Institute, Trinity College Dublin, Dublin, Ireland; Deutsches Krebsforschungszentrum, Germany

## Abstract

A novel single step assay approach to screen a library of photdynamic therapy (PDT) compounds was developed. Utilizing high content analysis (HCA) technologies several robust cellular parameters were identified, which can be used to determine the phototoxic effects of porphyrin compounds which have been developed as potential anticancer agents directed against esophageal carcinoma. To demonstrate the proof of principle of this approach a small detailed study on five porphyrin based compounds was performed utilizing two relevant esophageal cancer cell lines (OE21 and SKGT-4). The measurable outputs from these early studies were then evaluated by performing a pilot screen using a set of 22 compounds. These data were evaluated and validated by performing comparative studies using a traditional colorimetric assay (MTT). The studies demonstrated that the HCS assay offers significant advantages over and above the currently used methods (directly related to the intracellular presence of the compounds by analysis of their integrated intensity and area within the cells). A high correlation was found between the high content screening (HCS) and MTT data. However, the HCS approach provides additional information that allows a better understanding of the behavior of these compounds when interacting at the cellular level. This is the first step towards an automated high-throughput screening of photosensitizer drug candidates and the beginnings of an integrated and comprehensive quantitative structure action relationship (QSAR) study for photosensitizer libraries.

## Introduction

High content screening (HCS) is a powerful tool for the biological evaluation of potentially therapeutic compounds and widely used in drug discovery, biomedical research and pharmaceutical industry. This high throughput technique is based on high resolution microscopy and multi-parametric automated image analysis allowing a rapid quantitative evaluation of drug candidates on a large scale [1], [2]. It is particularly valuable during early drug development, and provides a physiologically relevant assay platform which utilizes intact cells [2], [3], [4]. This method allows a simultaneous detection of multiple biological pathways and the pre-clinical toxicological evaluation of pharmaceutical drugs. *In vitro* estimation of toxicity using HCS in cell lines has been used in recent years, particularly in predicting hepatic toxicity, but also to assess toxicity of anticancer agents [2], [3], [4], [5], [6], [7].

Photoactive compounds have found various biological and pharmaceutical applications in many areas including photomedicine. One of its branches includes photodynamic therapy (PDT), which is successfully used to treat different medical conditions including cancer for over 40 years [8], [9], [10], [11]. It is based on the accumulation of a photosensitizing drug (PS) in the target tissue, which generates toxic singlet oxygen and other reactive oxygen species upon irradiation with light [7]. As a non-invasive and non-scarring approach, PDT offers significant potential in cancer treatment. The majority of PS approved and under development drugs are porphyrin-type pigments [11], [12], [13], [14], [15]. Due to their biological significance and unique photophysical properties they found a variety of applications in several areas including PDT. Most of the photosensitizers used in cancer therapy have tetrapyrrolic structure similar to hematoporphyrins and significant attention has also focused on phytochlorin derivatives related to natural chlorophylls. As a result of the necessity to improve on the first and second generation of PSs, an enormous effort has been made by synthetic chemists worldwide. By now a multitude of new PSs have been synthesized and are currently under evaluation both *in vitro* and in clinical tests [13], [14]. However, *in vitro* studies on interactions of porphyrins and their derivatives with cells pose many difficulties. Even, natural light can activate these photoactive compounds leading to their photodegradation and also they may prematurely produce cellular damage. Therefore most experimental manipulations with the living cellular materials have to be carried out under special illumination conditions [7], [16], [17]. 16 By their very nature these compounds have an intrinsic fluorescence over a broad range of excitation and emission wavelengths hence making difficult to use conventional assays that are broadly used for a drug evaluation.

Note, that the *in vivo* toxicity of these PS is dependent on three factors: drug concentration and action, light and oxygen concentration. Thus any pharmacological investigation is more complex than that of ‘classic’ chemotherapeutic drugs. On the other hand the multimodal mode of action through reactive oxygen species mostly prevents resistance to develop.

Here we developed a HCS approach that can be used as a one-step assay to screen a library of PDT compounds. To attain a deeper understanding of how these compounds interact at the cellular level we complimented HCS output with quantitative structure-activity relationship (QSAR) studies; yet only few such studies have been reported for PDT and photosensitizer (PS) development [15], [16]. This is the first step towards an automated high-throughput screening of PS drug candidates and the beginnings of an integrated and comprehensive QSAR study for a library of photosensitizers.

## Materials and Methods

### Cell culture and treatment

The human esophageal squamous cell carcinoma cell line OE21 [17] and the human adenocarcinoma cell lines SKGT-4 [18] and OE33 [19], derived from Barrett’s esophagus, were purchased from the European Collection of Cell Cultures (ECACC).

All cell lines were cultured in RPMI 1640 (Hyclone, USA), supplemented with 10% inactivated foetal bovine serum (Hyclone, USA) and 1% Penicillin/Streptomycin (Hyclone, USA). Cultures were routinely grown in cell culture flasks (Nunc, Denmark) and split 1:8 until 70–80% confluency was reached. Cell recovery was achieved using 0.25% Trypsin with 0.1% EDTA in Hanks Buffered Salt Solution (Hyclone, USA) for three minutes at 37°C and 5% CO_2_ in humidified atmosphere and the culture medium was changed every three to four days until confluency was obtained.

For photodynamic treatment and uptake studies 3×103 OE21 cells per well were plated in 96-well plates (Nunc, Denmark) and treated with either 3 µM, 2 µM, 1 µM, or 0.5 µM of Temoporfin (*m*THPC) solution in the dark for up to 24 hours at 37°C and 5% CO_2_. Control cells were treated with vehicle alone. The Temoporfin and non-toxic glycoporphyrin solutions were prepared by dissolving 5,10,15,20-tetrakis(3-hydroxyphenyl)chlorin, synthesized following standard procedures [20], and 5,10-di(β−glucose–porphyrinato)zinc(II) derivatives in ethanol:propylene glycol = 3:2 (v/v) (Acros organics, USA). All other compounds tested were dissolved in neat dimethyl sulfoxide (DMSO) (Acros organics, USA) at 2 µM and the identified hits were then used to treat cells in the concentrations previously discussed. Vehicle controls containing the equivalent amounts of DMSO and the ethanol and propylene glycol mixture were also assessed for toxicity. A final screen of 22 new compounds dissolved in DMSO was performed in OE21, SKGT-4 and OE33 cell lines using a single concentration of 2 µM, for this OE33 and SKGT-4 cells were plated at a concentration of 6×103 cells per well and OE21 cells were used in the previously disclosed concentration. All potentially cytotoxic compounds identified in the two screens undertaken were prepared again at an initial concentration of 10 mM in DMSO and then tested at 24 µM, 12 µM, 6 µM, 3 µM, 2 µM, 1 µM and 0.5 µM in all cell lines.

### Illumination protocol

Following the removal of the medium with compound and addition of fresh, pre-warmed, fully supplemented RPMI the plates were separated into two groups. Group 1 was illuminated for two minutes using an illumination box, consisting of Luxeon High Power LEDs (LXHL-BW03) as light sources emitting white light with a total fluence rate of 1.7 mW.cm^−2^ [21]. Group 2 was kept in the dark under the same conditions as group 1. The cells were allowed to recover for four hours in the dark at 37°C and 5% CO_2_ in a humidified atmosphere.

### Cell proliferation assay

Two plates (one illuminated and one non-illuminated) were used for a 3-(4,5-dimethylthiazol-2-yl)-2,5-diphenyltetrazolium bromide (MTT) assay [22], [23] (Promega, USA), according to manufacturer’s instructions. Immediately, following illumination 15 µL of MTT dye was added to each well and kept for four hours at 37°C and 5% CO_2_ in a humidified atmosphere in the dark. The formatazan crystals were allowed to dissolve overnight at room temperature and absorbance was measured at 540 nm with a Wallac Victor2 plate reader (Perkin Elmer, Singapore).

Final dose-response experiments were performed using previously disclosed concentrations of compounds using the same plate however proliferation was assessed using a MTS kit (Promega, USA); these plates were allowed to recover 20 hours at which point of MTS previously combined with PMS dye was added according to manufacturer’s instructions. Plates were incubated for a further 4 hours then read at 490 nm with a Biotek EL800 (BioTek, USA).

### High content imaging and analysis

Following a four hour post illumination recovery two plates (one illuminated and one non-illuminated) were fixed with 4%, paraformaldehyde solution (Acros Organics, USA), pre-heated to 37°C C for 15 minutes and further stained with FITC-labeled phalloidin (Dyomics, Germany), an F-actin stain, and Hoechst (Invitrogen, USA), a nuclear stain. Images were collected using the InCell 1000 high content system (GE Healthcare, USA). A total of ten fields per well were imaged under 10× magnification using three separate filters to capture the nucleus (blue, excitation 345 nm, emission 435 nm), F-actin (green, excitation 475, emission, 535 nm) and Temoporfin® (red, excitation 560 nm, emission 700 nm). In order to obtain information about cell number a further seven fields (covering the vast majority of the well) were obtained at 4× magnification using a single blue filter, collecting nuclear fluorescence alone.

Image analysis was performed using the InCell 1000 image analyzer (GE Healthcare, USA) using three different algorithms according to the parameters needed. In order to determine cell and nuclear area a morphology algorithm was used, while for photosensitizer parameters a dual area object analysis algorithm was used and a multi target analysis algorithm was used in a separate set of images (4× objective) in order to determine cell number (Table 1).

**Table 1:** Description of parameters used in the image analysis performed using the InCell investigator software.

Parameter	Description
Nuclear area	Area of identified cell nucleus
1/form factor	Mean cell roundness index. Values range from 1 to infinity where 1 is a perfect circle (elongation of cell)
Cell area	Area of identified cell body
Cell number	Number of identified cell nuclei
PS integrated intensity	Average fluorescence intensity of pixels within the cytoplasmic region of the red PS multiplied by the area of identified red fluorescence
PS area	Area of identified intracellular red fluorescence

The image analysis software detects cells nuclear area/cell number by nuclear dye uptake with quantifications of cell morphologies and PS parameters determined by F-actin stain and *m*THPC. Stain parameters such as morphology and intensity of fluorescence were logged numerically for individual cells in every field as well as averages for each field and well [24].

### Statistical analysis

Each experiment was repeated a minimum of three times and results were normalized to untreated controls. Averages and standard error of the mean (SEM) of each well were plotted using Graphpad Prism version 5.0 (GraphPad Software, USA). Differences in the cell proliferation data were evaluated by ANOVA analysis with Bonferroni comparison. Correlation between InCell and MTT data was evaluated by Pearson correlation test with a 99% confidence. Regressions (linear and non-linear) were fitted to the photosensitizer data (intensity and area). All statistical analyses were performed using Graphpad Prism version 5.0.

## Results and Discussion

Automated imaging has been available for the last 20 years and it has significantly streamlined the process of *in vitro* drug screening in addition to decreasing the time needed both for high quality image collection and analysis [24], [25]. Naturally fluorescent drugs have, in the past, been evaluated by fluorescent microscopy [26], [27], [28], [29]; however, a phenotypic screen of toxicity in cells using naturally fluorescent compounds has seldom been attempted due to its inherent restrictions. In order to overcome these apparent limitations here we present the results obtained for a novel high content assay developed using fluorescent drugs in OE21 cells, a human esophageal squamous cell carcinoma cell line derived from a human carcinoma. Also, we present two test screens with novel photosensitizers in three different esophageal cell lines, the above mentioned OE21 cells, SKGT-4 and OE33 cells, a human adenocarcinoma of the esophagus derived from a human adenocarcinoma caused by Barrett’s esophagus.

As a reference compound we chose Temoporfin (5,10,15,20-tetrakis(3-hydroxyphenyl)chlorin, *m*THPC] as PS. This drug is an approved PS for the treatment of head and neck malignancies (*e.g.*, known in one formulation as Foscan®) and is widely used in developmental studies and investigations on other cancer types [20], [30], [31]. Thus, this classic PS is a suitable positive control for screening and assay development. In line with our interest in malignancies of the GI (gastrointestinal) tract, we chose three esophageal cancer cell lines (OE21, SKGT-4 and OE33), which we have used extensively for *in vitro* studies. OE21 is a squamous cell carcinoma of the esophagus that is commercially available and is widely used as an esophageal cancer *in vitro* model [32], [33]. SKGT-4 is a well differentiated adenocarcinoma of the esophagus cell line, derived from Barrett’s epithelium [18] and used worldwide in cancer studies, particularly those focused on Barrett’s epithelium [33], [34]. OE33 is a commercially available adenocarcinoma of the lower esophagus cell line derived from Barrett’s metaplasia [19].

### Cell proliferation

**Figure 1:**
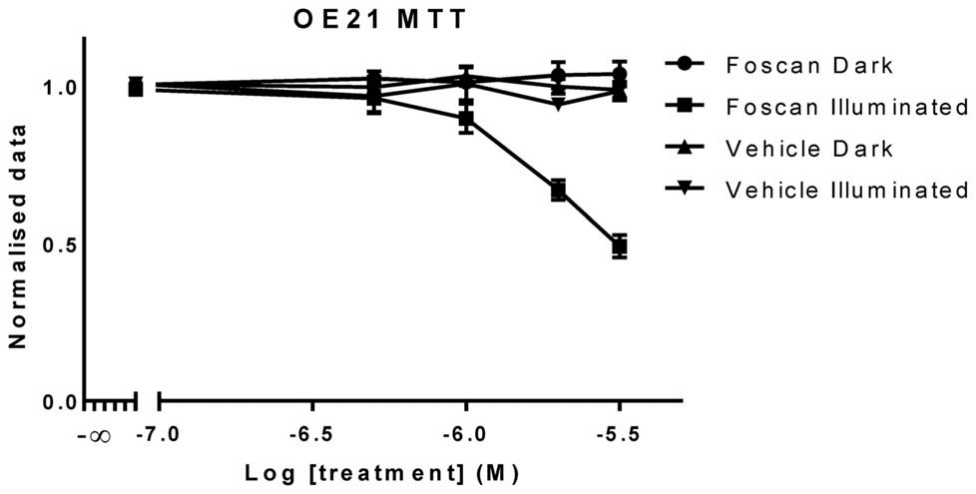
OE21 cells treated with increasing concentrations of *m*THPC and with equivalent concentrations of vehicle alone (illuminated and non-illuminated) were assessed for toxicity using a traditional cell proliferation assay (MTT). Data are representative of independent experiments and values are expressed in mean ± SEM.

MTT measures the proliferation of cultures through an estimation of mitochondrial activity; this is achieved by the reduction of MTT into formatazan [22], [23]. The reduction is performed by the action of mitochondrial dehydrogenases in metabolically active cells; the amount of formatazan produced is known to be directly proportional to the mitochondrial enzymatic activity and, therefore, to the number of proliferating cells in culture [22], [23], [24], [25]. This assay has been extensively used both to determine cell proliferation, viability and to determine toxicity of potential therapeutic agents [35], [36], [37]. First, the effect of *m*THPC on OE21 cells was tested with the MTT test in order to have a classical toxicity result with a widely used technique. As shown in Figure 1 there is a decrease in cell viability upon plate illumination with increasing *m*THPC concentrations. No dark or vehicle toxicity was observed. The two way ANOVA analysis showed a statistically significant difference between illuminated and non-illuminated data where *m*THPC treated cells showed a statistically significant difference from concentration 2 µM up. The same statistical analysis showed that vehicle treated cultures showed no such difference in the presence or absence of light. These results are in accordance to the literature where *m*THPC alone or vehicle were shown to have no effect but *m*THPC treated cells in the presence of light showed significant toxicity in a dose dependent manner [20], [30].

### High content analysis

**Figure 2:**
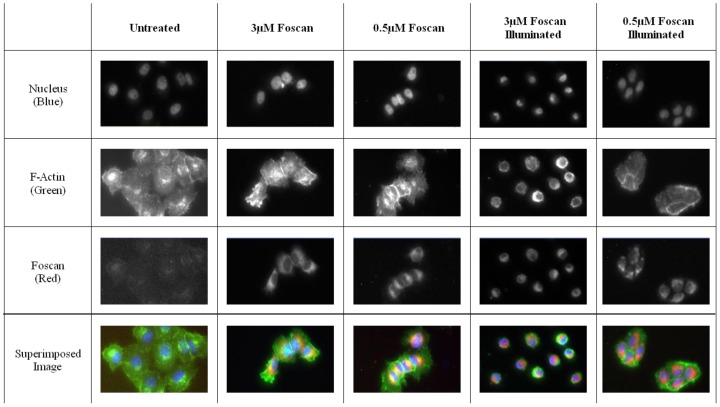
Examples of images collected with the InCell imaging system for the different treatments used with OE21 cells treated with 3µM Temoporfin for 24 hours before illumination and fixed at 4 four hours post illumination in 4% paraformaldehyde. Cells were labeled with Hoechst–nucleus (blue at 345 excitation, 435 emission), phalloidin–F-actin (green at 475 excitation and 535 emission), *m*THPC (red) was aquired at 560 excitation and 700 emission. Images were acquired by an InCell Analyzer automated microscope using a 10x objective (image size 0.897 mm×0.671 mm).

Next, we used an HCA platform to analyze the morphological changes that occur upon PDT treatment of OE21 cells. Cell death results in many changes of cellular morphological characteristics and these may be used for HCA analysis. Figure 2 shows some examples of images collected using the HCA platform of OE21 cells. Clearly, illumination in the presence of the photosensitizer results in dramatic cellular changes compared to the dark control. The severity of these physical cell deformations increased with the *m*THPC concentration.

**Figure 3:**
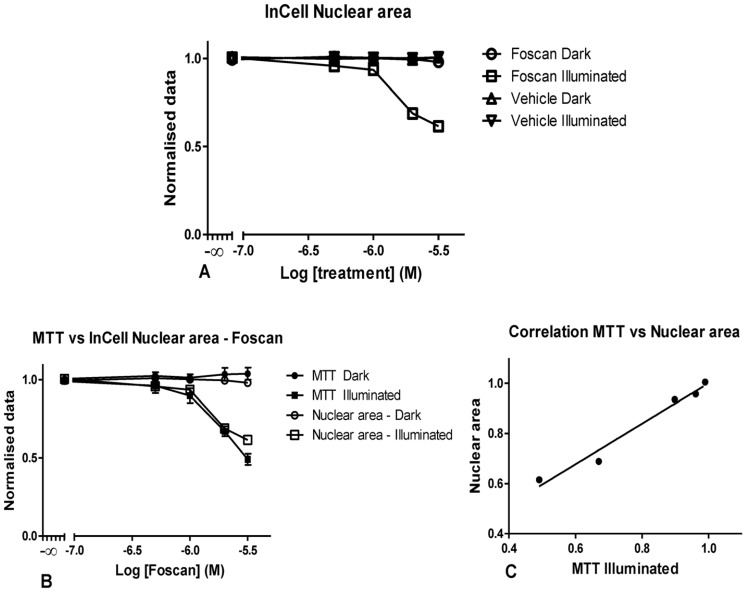
Graphical representation of the nuclear area data generated using the InCell image analysis in OE21 cells. Representation of the nuclear area with increasing concentrations of *m*THPC and with equivalent concentrations of vehicle in illuminated and dark cells (A). Data generated by MTT were compared to HCS data (B) for illuminated cells in order to establish the degree of correlation (C). Data are representative of four independent experiments and values are expressed in mean ± SEM.

The earliest phenotypic signs of cell toxicity are reflected by changes in nuclear size, cell fragmentation, and significant changes in cell area and a cell shape [38]. Other phenotypic changes include cell membrane breakdown, leakage of cellular contents and increased numbers of cytosolic, lysosomal vesicles. Clearly an automated approach to the quantification of these features can be useful during a screen of large numbers of compounds by taking advantage of these marked changes in cell morphology. Typically, changes in nuclear morphology are amongst the first effects seen during cell death [39]. The results show that the nuclear area in OE21 cells in fact reflected the phototoxicity of Temoporfin (Fig. 3). Not only does nuclear area reflect toxicity in a dose-dependent fashion (Fig. 3A), where it is clear that nuclear area was reduced with increasing concentrations of Foscan, and followed this trend in the same fashion as seen in the cell proliferation assay (Fig. 3B). The two way ANOVA analysis revealed that when comparing dark nuclear area and illuminated nuclear area there was a significant difference in the data up to, but not including, the 1 µM dose. On the other hand, comparison of MTT in the dark with illuminated MTT data showed a significant difference in the data up to, but not including, 1 µM of *m*THPC.

A comparison of MTT in the dark with nuclear area in the dark, showed no statistically significant difference in the data at any of the concentrations tested. However, a comparison of their illuminated counterparts indicated a significant difference in the data at 3 µM. This difference was not observed at any other concentration. In order to evaluate if the trends observed in the nuclear area data and MTT data were related, a Pearson correlation test was performed. This confirmed a very high correlation between the MTT results and the nuclear area with P = 0.0009 with a 99% confidence interval between 0.59 and 0.99 (Fig. 3C). There is also a clear decrease in data variability as indicated by an absence or reduction of the SEM bars in Fig. 3A.

**Figure 4:**
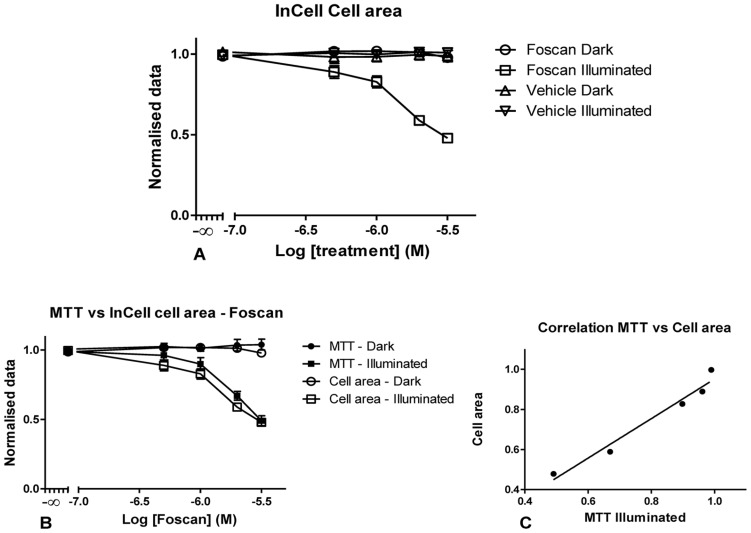
Graphical representation of the cell area data generated using the InCell image analysis (A and B) using OE21 cells. Representation of the cell area with increasing concentrations of *m*THPC and with equivalent concentrations of vehicle illuminated and dark cells (A). Data generated by MTT were compared to HCS data (B) for illuminated cells in order to establish the degree of correlation (C). Data are representative of four independent experiments and values are expressed in mean ± SEM.

Another morphological parameter to change upon PDT treatment is the cellular area (Fig. 4). Note, that the images in Fig. 1 indicated that the cell area was severely affected by the action of *m*THPC and light. For OE21 cell cultures the graphical representation of the data showed a significant reduction of cell area with increased concentrations of Temoporfin, yet, no differences were observed in cells treated with vehicle alone or those kept in the dark (Fig. 4A). This particular parameter showed the same trend as the classical MTT assay (Fig. 4B) and seems to be slightly more sensitive at detecting toxicity. Again, there is a very high correlation between the data generated by detecting cell area and the MTT assay (Fig. 4) with P = 0.0014 with a 99% confidence interval in the 0.49 to 0.99 range (Fig. 4C). The ANOVA test showed no statistically significant difference between illuminated to dark cell area at all the *m*THPC concentrations tested. Conversely, there were no statistically significant differences between MTT and cell area regarding cultures kept in the dark, and the same result was obtained when testing illuminated cultures.

Other morphological parameters were investigated, yet failed to yield any informative data in this particular cell line. Among these were nuclear displacement, shape, roundness, cell elongation, and intensity spreading. While the images obtained qualitatively indicated that these parameters could reflect toxicity this could not be confirmed in a statistically reliable manner. All measurements parameters indicated that 2 µM of *m*THPC was sufficient to give a 50% inhibition in response; this IC50 was obtained by fitting a dose response non-linear regression with variable slope to the data. This value was found to be consistent between all HCA parameters analyzed and well as the MTT assay performed.

**Figure 5:**
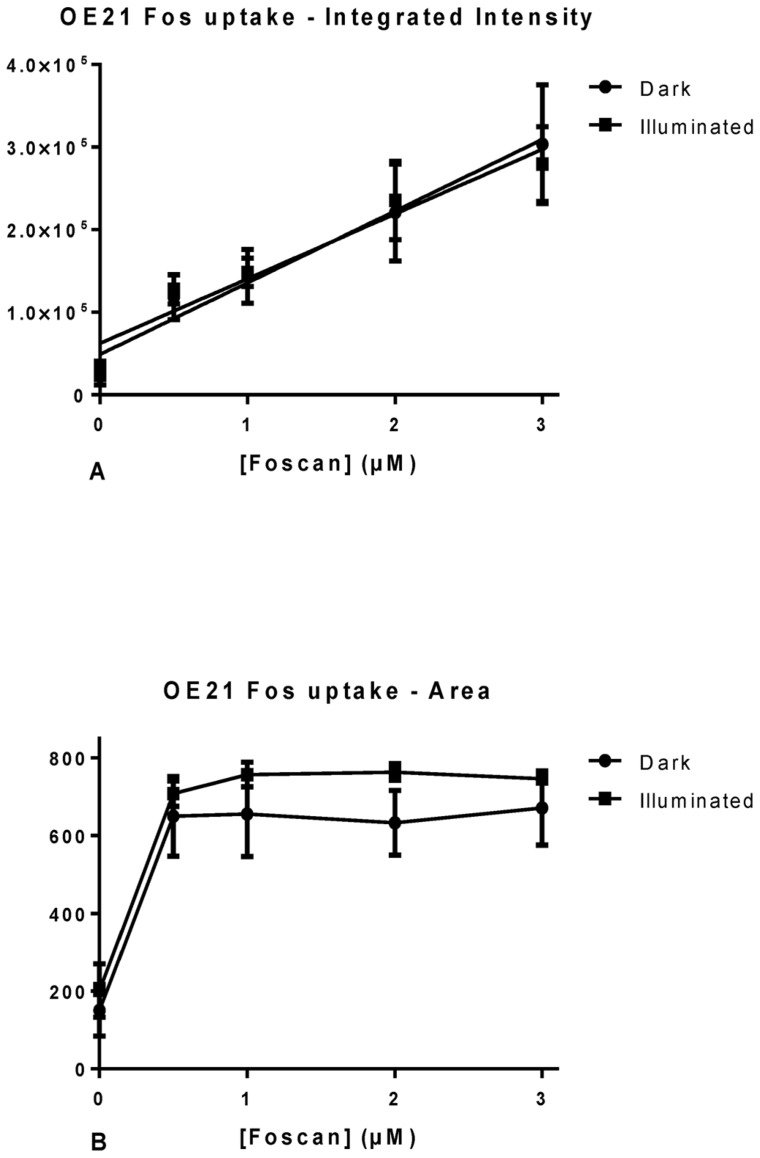
Graphical representation of the results obtained from InCell analysis for photosensitizer parameters. Intracellular integrated intensity (A) and area (B) in OE21 cells. Data are representative of three independent experiments and values are expressed in mean ± SEM.

**Figure 6:**
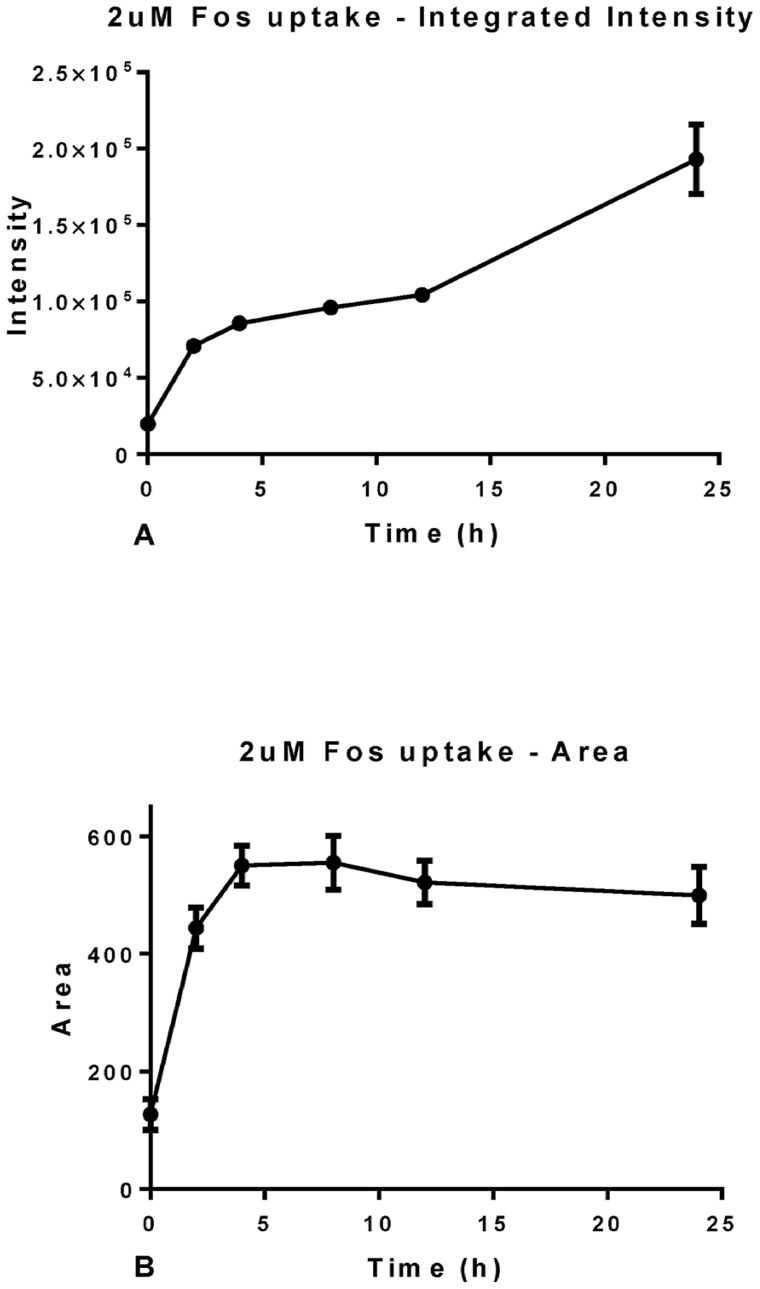
Graphical representation of the results obtained from InCell analysis for photosensitizer (2µM) uptake in OE21 cells. Intracellular intensity (A) and area of the photosensitizer (B); we followed OE21 cultures incubated with *m*THPC for various times. Data are representative of three independent experiments and values are expressed in mean ± SEM.

A significant advantage of using suitable PDT related porphyrins with HCA is the inherent fluorescence of the drugs. While this is essential for the desired intra- and extracellular action it also provides an extremely valuable information tool. Compared to the HCA of non-fluorescent drugs no staining or processing of the cultures is needed to access information. For example, as shown in Fig. 5 and Fig. 6, PS fluorescence can be used to directly monitor photosensitizer uptake. Again, using the HCA platform two parameters were utilized for the analysis: intracellular integrated intensity (Fig. 5Aand 6A) and area of the photosensitizer (Fig. 5B and 6B).

Intensity measurements showed that there was an increase in intensity with increased concentrations of Temoporfin and this was not significantly affected by illuminating the culture (Fig. 5A). This was assessed by means of student *t*-test where P = 0.87 with 99% confidence interval of −214013 to 218817. Linear regressions fitted to the OE21 cell data showed that in both illuminated and dark cultures there was a significant deviation from zero, with r^2 = ^0.66 with P = 0.0002for dark cultures and r^2 = ^0.74 with P<0.0001 for illuminated cultures (Fig. 5A).

For OE21 cells, the *m*THPC intracellular area exhibited a linear increase of occupied area within the cell up to 1 µM of dosage; any higher dose did not yield a higher area within the cells (Fig. 5B). This suggests that the PS is occupying a finite area within the cell; as even though the area occupied by *m*THPC stabilizes the fluorescence intensity continues to increase. This is in agreement with published results on the localization of the drug in the Golgi apparatus and the endoplasmatic reticulum [40], [41], [42], [43].

Using 2 µM of *m*THPC its uptake was tracked with time for up to 24 hours (Fig. 6). Following the fluorescence intensity with time revealed a linear increase of integrated intensity up to 24 hours of incubation (Fig. 6A). The linear regression fitted to the data showed a significant deviation from zero with r^2 = ^0.85 and P<0.0001 (Fig. 6A). This result is in accordance to the literature, where uptake of *m*THPC was found to be optimum at 24 hours for other cell lines [30]. Also, an incubation period of 24 hours yielded a higher uptake of the PS than any of the other times tested and thus could yield higher toxic effects. Initially, a linear increase in PS area from 0 to 5 hours was noted and then the area stabilizes (Fig. 6B). Thus, cells should be incubated for a minimum time of five hours prior to illumination.

**Figure 7:**
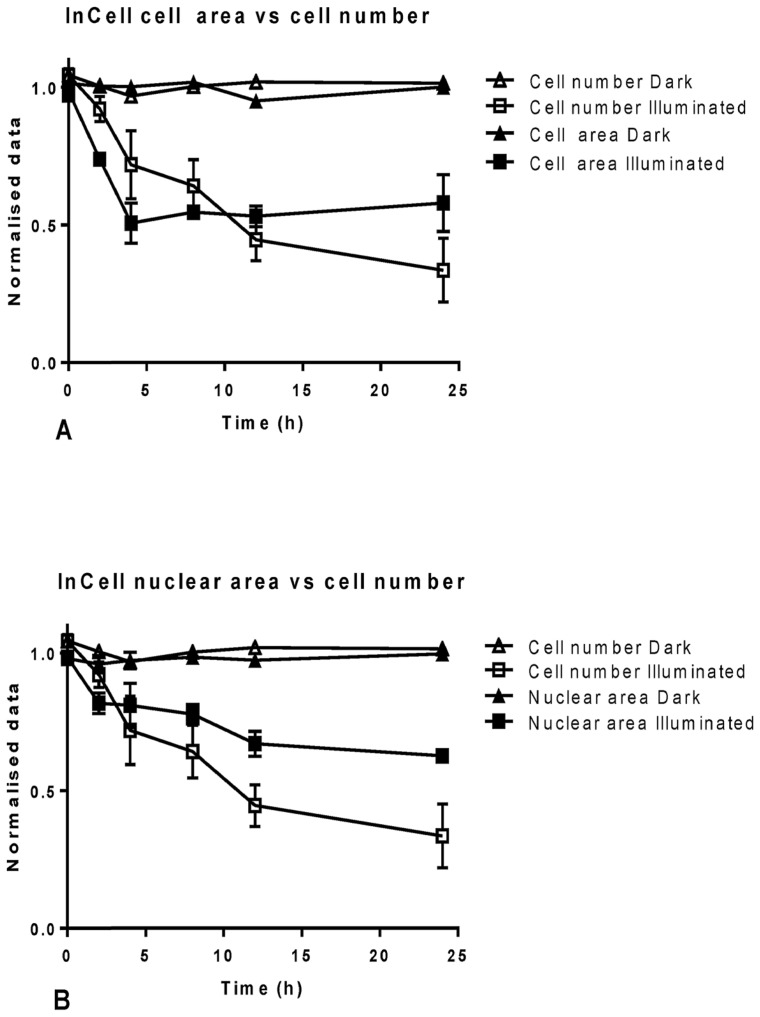
Graphical representation of the results obtained from InCell analysis for OE21 cultures treated with 2 µM *m* THPC and followed for up to 24 hours. Cell number was compared to cell area over time (A) and to nuclear area over time (B). Data are representative of three independent experiments and values are expressed in mean ± SEM.

Cell proliferation is directly dependent on cell health and impairment thereof affects the cell number [44], [45]. Thus, monitoring of the cell number over time could be an important marker of culture health and, consequently, could be used to predict toxicity of possible therapeutics in an *in vitro* assessment. Thus, OE21 cell cultures were incubated with 2 µM of *m*THPC, the cell numbers were determined and compared to nuclear and cellular area over the course of 24 hours (Fig. 7). The results showed that non-illuminated cells did not exhibit a change in any of the parameters over time (Fig. 7A and 7B). On the other hand, cell area decreased in a linear fashion from 0 to 4 hours and then stabilized. However, when this parameter stabilized the cell number began to decrease significantly from 2 to 24 hours (Fig. 7A). In the same fashion, nuclear area decreased steadily from 0 to 12 hours and then stabilized. Again, at this time, the cell number continued to decrease in a linear fashion (Fig. 7B). This clearly illustrates the cell number to be a more reliable marker when tracking a culture over time.

Using the High Content Analysis we have identified several cellular parameters which were validated against the MTT assay. Of these parameters the most robust were nuclear and cellular area. In addition, it is possible to obtain detailed information on the concentration dependency of PS in living cellular systems from both intensity and area occupied by *m*THPC within the both types of cell lines tested in a single experiment. The new assay reliably validated that light, vehicle, and *m*THPC alone did not induce cell death and reproduced the difference between illuminated and non-illuminated cells treated with the PS. Similarly, it was possible to follow PS uptake in a time and concentration dependent manner; especially when using the cell number as a measure of toxicity.

### High content analysis of a library of porphyrins

**Figure 8:**
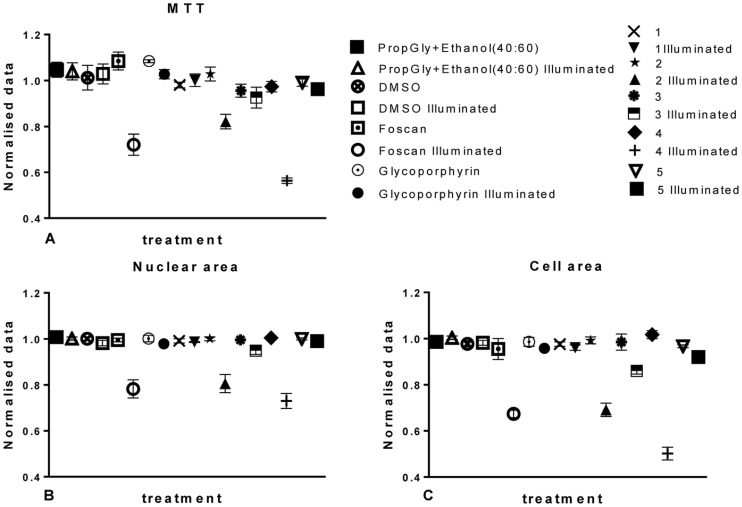
Graphical representation of five porphyrins and respective controls at 2µM concentration alone. MTT data (A) was compared with the previously selected HCA parameters, nuclear area (B) and cell area (C). Data are representative of three independent experiments and values are expressed in mean ± SEM.

**Figure 9:**
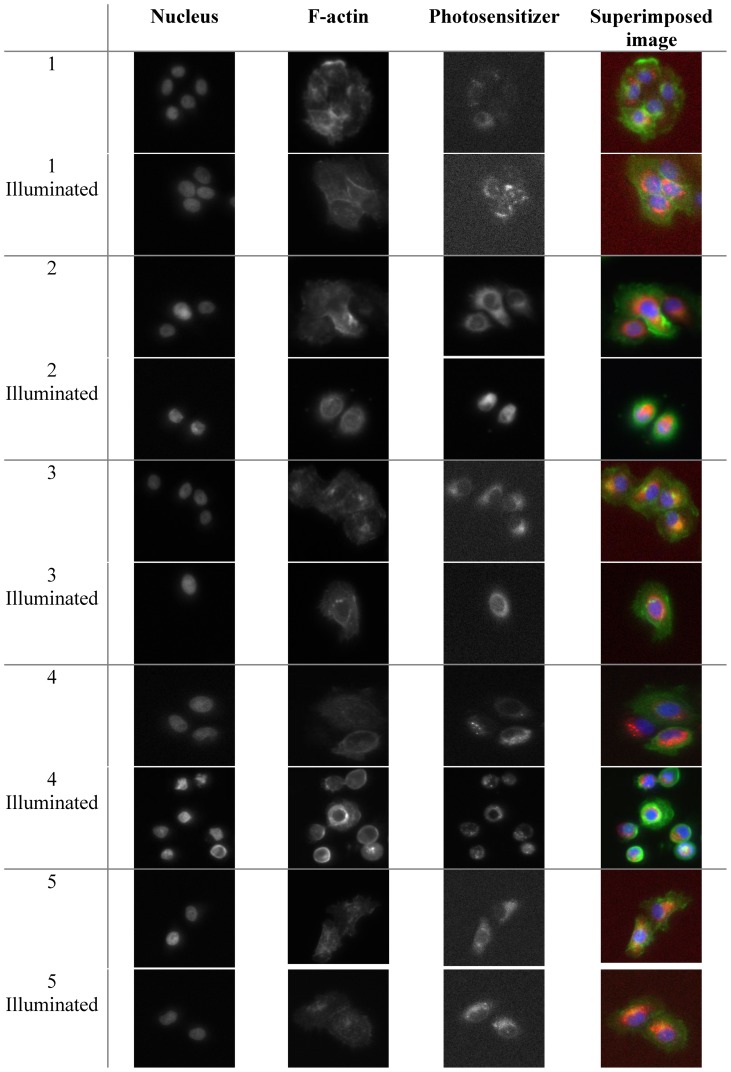
Examples of images collected with the InCell imaging system for the different treatments used with OE21 cells treated with 3 µM of five different potential photosensitizers for 24 hours before illumination and fixed four hours post illumination in 4% paraformaldehyde. Cells were labeled with Hoechst–nucleus (blue at 345 excitation, 435 emission), phalloidin–F-actin (green at 475 excitation and 535 emission), PS (red) weres aquired at 560 excitation and 700 emission. Images were acquired by an InCell Analyzer automated microscope using a 10x objective (image size 0.897 mm×0.671 mm).

In order to further validate our hypothesis, we initially tested a panel of five porphyrins using both methodologies in OE21 cells using a single concentration of 2 µM (Fig. 8). The five compounds studied were: **1**–5-(4-methylphenyl)-10-phenyl-15-(2,4,6-trimethoxyphenyl)porphyrin; **2**–*m*THPP [5,10,15,20-tetra(3-methoxyphenyl)porphyrin], the parent porphyrin of *m*THPC; **3**–5-(4-hydroxyphenyl)-10,20-diphenylporphyrin; **4**–7-demethyl-7-formyl-chlorin e_6_; **5**–5-(4-benzoic acid)-10,20-di(3-methoxyphenyl)porphyrin. The one way ANOVA results of these experiments with Bonferroni's Multiple Comparison Test showed no difference between any of the vehicles tested (either illuminated or in the dark); only compounds **2** and **4** showed any effect upon illumination (Fig. 8). Thus, dose-response studies (both MTT and HCA) were performed with these hits. The results were visually confirmed from the HCA images. These show that, in fact only cultures treated with *m*THPC and **2** and **4** and subsequently illuminated showed any morphological changes (Fig. 9).

**Figure 10:**
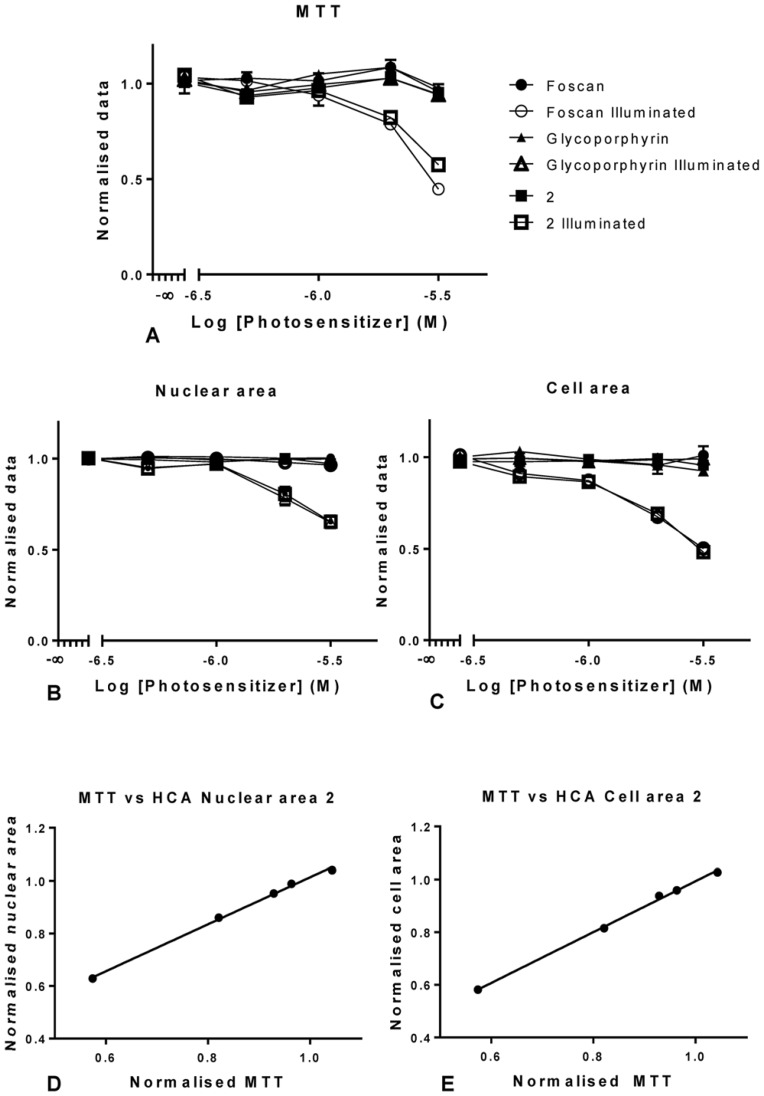
Graphical representation of the data for porphyrin 2, using MTT (A) and the InCell image analysis (B and C) in OE21 cells. Representation of the nuclear area with increasing concentrations of *m*THPC and porphyrin 2 (B) and the equivalent for cellular area (C) in illuminated and dark cells (A and B). Data for illuminated cells were compared to establish the degree of correlation with both HCA parameters tested (D and E). Data are representative of three independent experiments and values are expressed in mean ± SEM and linear regressions were fitted (A, B and C).

We tested various concentrations of the porphyrin **2** (Fig. 10), and compared the results to *m*THPC as positive control, and to a non-phototoxic glycoporphyrin as negative control. The HCA method was used, and validated by MTT, to determine the toxicity of **2** within the previously tested concentration range (Fig. 10). The results show that **2** followed a similar pattern of action as *m*THPC. This is not too surprising as **2** is the porphyrin analogue of the Foscan PS [44]. Overall, the MTT evaluation of **2** (Fig. 10A) confirmed the data from the HCA study. No differences were found between **2** and *m*THPC in the dark; for the illuminated cells a difference was only found at 3 µM alone. Similar results were found for the nuclear area (Fig. 10B) and a high correlation between nuclear area and MTT data was shown by a Pearson correlation; with P<0.0001 with a 99% confidence interval from 0.95 to 1 (Fig. 10D).

The cell area plot (Fig. 10C) showed significant differences only when comparing *m*THPC treated cells kept in the dark to those that were illuminated from, but not including 0.5 µM. Similar results were obtained cells treated with 2 in the dark and compared to illuminated ones. The results suggest that cell area is a more sensitive parameter at detecting toxicity. Conversely, there were no statistically significant differences between any of the other parameters tested. The correlation of the HCA data with the MTT assay was, again, significant with P<0.0001 with a 99% confidence interval from 0.96 to 1 (Fig. 10E). The concentrations of vehicles used were also tested and showed no toxicity (data not shown).

**Figure 11:**
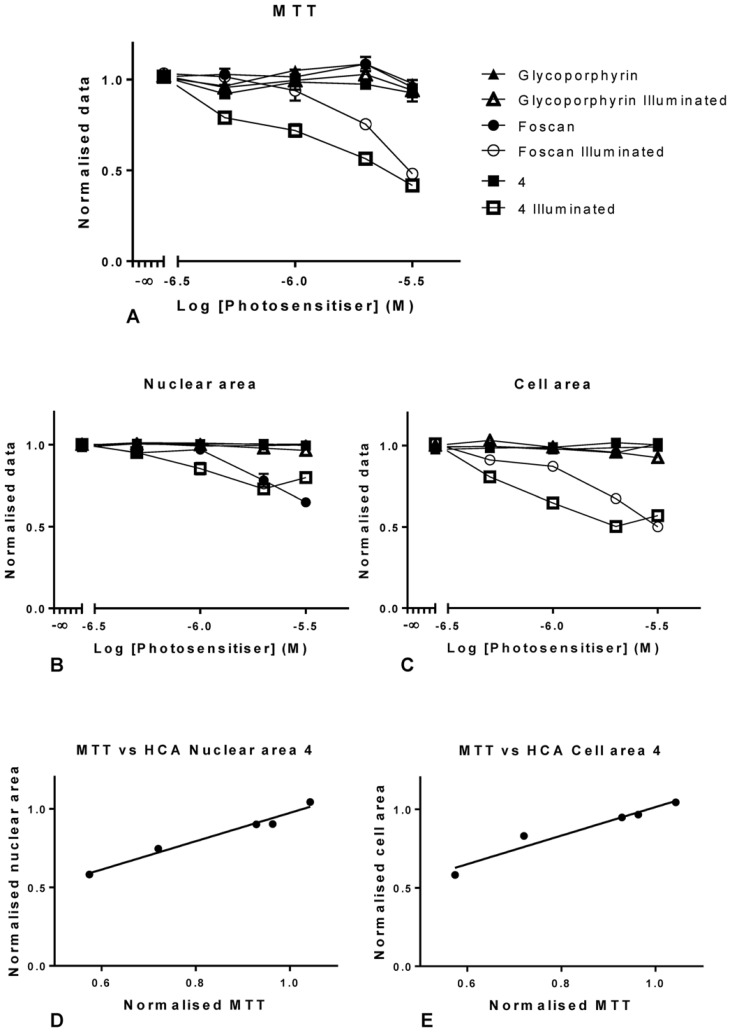
Graphical representation of the data generated for rhodochlorin 4, using MTT (A) and the high Content image analysis (B and C) in OE21 cells. Representation of the nuclear area with increasing concentrations of *m*THPC and chlorin 4 (B) and the equivalent for cellular area (C) in illuminated and dark cells (A and B). Data generated for illuminated cells were compared to establish the degree of correlation with both HCA parameters tested (D and E). Data are representative of three independent experiments and values are expressed in mean ± SEM and linear regressions were fitted (A, B and C).

The initial screening data in OE21 cells indicated that compound **4** exhibited a more powerful inhibitory effect than the positive control, *m*THPC (Fig. 11A). As a rhodochlorin derivative this agrees with expectations, as such systems have been shown to be highly suitable PS [11]. The MTT evaluation of **4** (Fig. 11A) showed a statistical significant difference between Temoporfin treated cultures and those treated with **4** at 2 and 0.5 µM concentrations. A detailed analysis of cells treated with **4** and those treated and illuminated showed significant differences at all concentrations tested. In fact, a comparison of illuminated *m*THPC cells *versus* **4** illuminated cultures gave statistically significant differences throughout all concentrations tested (Fig. 11A). Thus, **4** has a stronger inhibitory effect than *m*THPC against OE21 cells. All other results were as expected; no difference was found between dark and illuminated cultures treated with the control, non-phototoxic glycoporphyrin.

Similarly, the same results were obtained for the nuclear area (Fig. 11B) and a high correlation between nuclear area and MTT data was confirmed by a Pearson correlation with P = 0.0057 with a 99% confidence interval from 0.29 to 0.99 (Fig. 11D). Similar to the studies with **2**, cell area upon treatment with **4** was found to reflect toxicity in reliable manner. In this case, the correlation of the HCA data with MTT was again significant with P = 0.017 with a 99% confidence interval from 0.61 to 0.99 (Fig. 11E).

**Figure 12:**
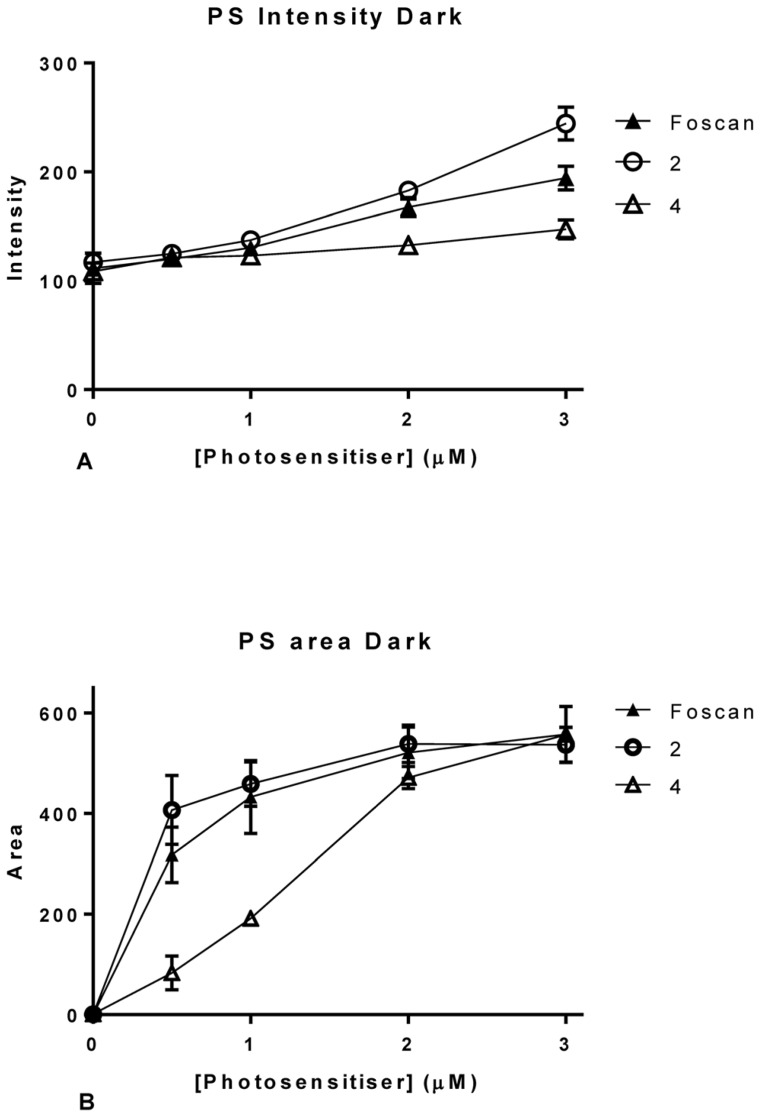
Graphical representation of the results obtained from InCell analysis for photosensitizer parameters in OE21 cells; intracellular intensity (A) and area of the photosensitizer (B). Data are representative of three independent experiments and values are expressed in mean ± SEM.

As shown above, intensity measurements gave an increase in intensity with increasing concentrations of *m*THPC (Fig. 12A). This was confirmed for both test compounds (Fig. 12A). ANOVA analysis revealed **2** to be different from *m*THPC at 3 µM while **4** differed significantly at 2 and 3 µM. This can be visually confirmed by the graphical representation where it was observed that **2** has a slightly higher intensity than *m*THPC, probably due to less fluorescent quenching of this compound in the cells. Compound **4** on the other hand showed less fluorescence than Temoporfin in the cells. In *m*THPC treated cells, intracellular area exhibited the expected linear increase of area within the cell up to 1 µM of dosage (Fig. 12B) and the same pattern was observed for **2**. For **4** a linear relationship with increasing concentrations was found. However, the area occupied by this compound in the cells treated with 2 and 3 µM was the same as in Foscan treated cells, as validated by ANOVA. Putatively, the two compounds have different uptake mechanism.

A final screen of 22 compounds was performed in three cell lines (OE21, SKGT-4 and OE33) using a single concentration of 2 µM. In order to evaluate our analytical strategy for comparative biological evaluation we decided to investigate a series of porphyrin photosensitizers with a varied degree of photoactivity. The compounds have been divided into the three sub-groups consisting of hemes, pheophorbides, porphyrins and ABCD-type structures [46], [47].

The compounds analyzed were: **6**–haematoporphyrin; **7**–hemin; **8**–methyl pheophorbide b; **9**–glucose pentaacetate; **10**–5-(3,6-dimethoxyphenyl)-2,3,7,8,12,13,17,18-octaethyl-10,15,20-triphenylporphyrin; **11**–5-bromo-15-hexyl-10,20-bis(3-hydroxyphenyl)porphyrin; **12**–(*E*)-3-[(5,10,15-triphenylporphyrinato-20-yl)nickel(II)]acrylic acid; **13**–3-hydroxymethyl-4-nitrophenol; **14**–(2-(2-chloroethyl)-17-ethyl-3,7,13,18-tetramethyl-12-methoxycarbonyl-8-(propionic acid methyl ester)porphyrinato)zinc(II); **15**–methyl pheophorbide a; **16**–(*E*)-methyl 4-(5,10,15-triphenylporphyrin-20-yl)but-2-enoate; **17**–(2*E*,2′*E*)-dimethyl 4,4′-[5,15-triphenylporphyrin-10,20-diyl]dibut-2-enoate; **18**–5-(4-benzoic acid)-15-hexyl-10,20-bis(3-hydroxyphenyl)porphyrin; **19**–methyl 5-(4-benzoic acid)-15-hexyl-10,20-bis(3-hydroxyphenyl)porphyrin; **20**–5-(2-cyanoethyl)-10,15,20-tris(4-methylphenyl)porphyrin; **21**–(5,10,15-triphenylporphyrinato)zinc(II); **22**–(2-(2-chloroethyl)-17-ethyl-3,7,13,18-tetramethyl-12-methoxycarbonyl-8-(propionyl piperidine amide)porphyrinato)zinc(II); **23**–5-(4-ethinylphenyl)-10,20-diphenylporphyrin; **24**–protoporphyrin IX dimethyl ester; **25**–(2,3,7,8,12,13,17,18-octaethyl-5-nitro-porphyrinato)zinc(II); **26**–[5-hexyl-10,20-diphenyl-15-(spirobis-1,3-dithian-2-yl)porphyrinato]nickel(II); **27**–chlorin e_6_ trimethyl ester. From these experiments we were able to determine that non-illuminated cultures showed no toxicity (data not shown).

**Figure 13:**
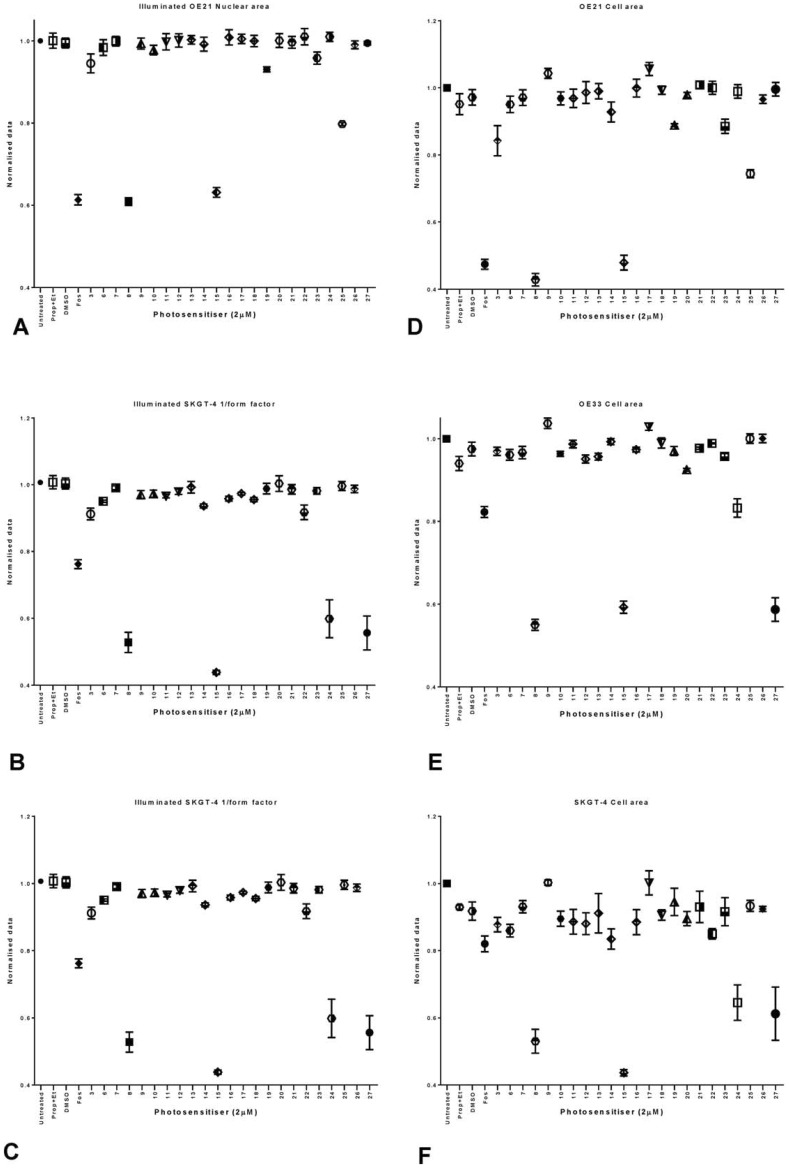
Graphical representation of the results obtained from InCell analysis for a library of 22 different compounds tested in OE21 cells (A) OE33 cells (B) and SKGT-4 cells (C). In terms of nuclear area (A, B and C) and cell area (D, E and F). Data are representative of three independent experiments and values are expressed in mean ± SEM.

However when cultures were illuminated we found that only compounds **8**, **15**, **24** and **27** showed activity in all cell lines (Fig. 13). Nuclear area, however, failed to reveal any relevant toxicity information in SKGT-4 cells as it mostly remained unchanged (data not show). Nonetheless, the large number of output parameters from the image analysis indicated that the parameter 1/form factor can yield significant information about the toxicity of *m*THPC and the test compounds in SKGT-4 cells. This cell line differs much in appearance to OE21 and OE33 cells. They have a more stretched appearance and the phenotype changes significantly upon treatment with Temoporfin; the cells became much more round in shape than their untreated and non-illuminated counterparts. The results show that the 1/form factor in SKGT-4 (Fig. 13C) cells in fact reflects the phototoxicity of Temoporfin and compounds **8**, **15**, **24** and **27** in a similar fashion as does the nuclear area for OE21 (Figure 13 A) and OE33 cells (Fig. 13B). No toxicity was observed upon treatment with vehicle alone (Fig. 13A, B and C). These results were confirmed by the cell area analysis for all three analyzed cell lines (Fig. 13 D, E and F). Of note is the fact that different cell lines seem to respond differently to the different compounds tested.

**Figure 14:**
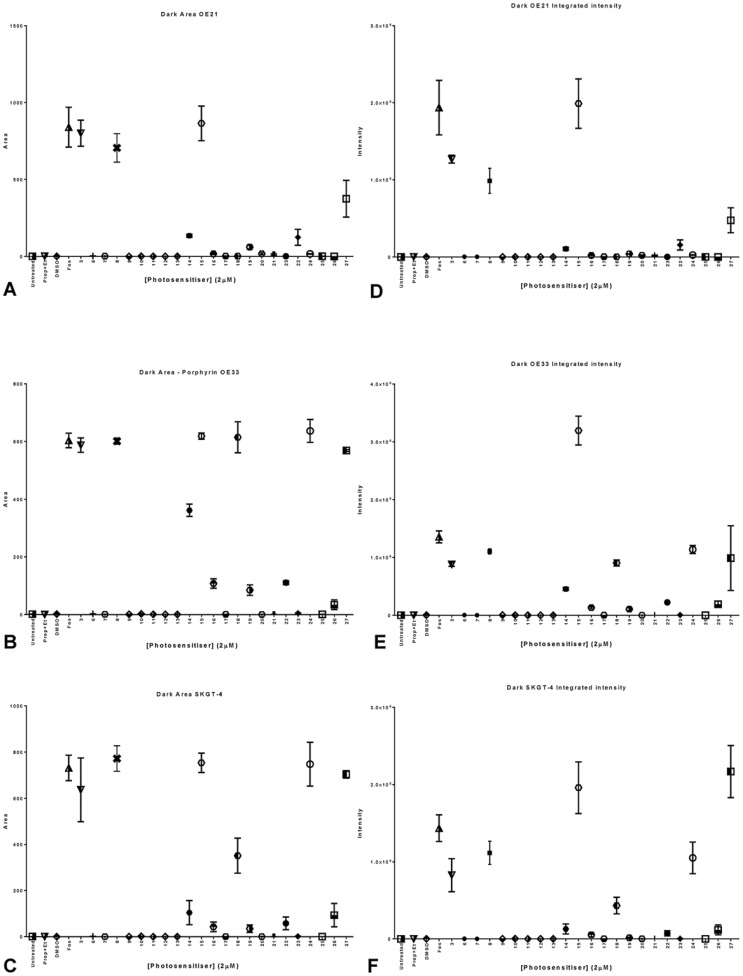
Graphical representation of the results obtained from InCell analysis for a library of 22 different compounds tested in OE21 cells (A) OE33 cells (B) and SKGT-4 cells (C). In terms of integrated intensity of the potential photosensitizers (A, B and C) and area (D, E and F). Data are representative of three independent experiments and values are expressed in mean ± SEM.

From the compound parameters (integrated intensity and area) we could determine that from the 22 compounds tested only **8**, **14**, **15**, **18**, **22**, **24** and **27** were taken up by the cells (Fig. 14 C, D and E) this explains many of the results obtained for the activity output, in fact most compounds are not taken up by the cells at all. From our results we can also deduce that compounds **8**, **15**, **18** and **24** (Fig. 14 A, B and C) occupy a similar intracellular space as *M*thpc, but compounds **14**, **22** and **27** occupy a different area or the cells (Fig. 14A, B and C). The previously discussed trend where integrated intensity seems to be directly related to effectiveness of the compound is maintained in this larger primary screen (Fig. 13 and Fig. 13 C, D and E).

**Figure 15:**
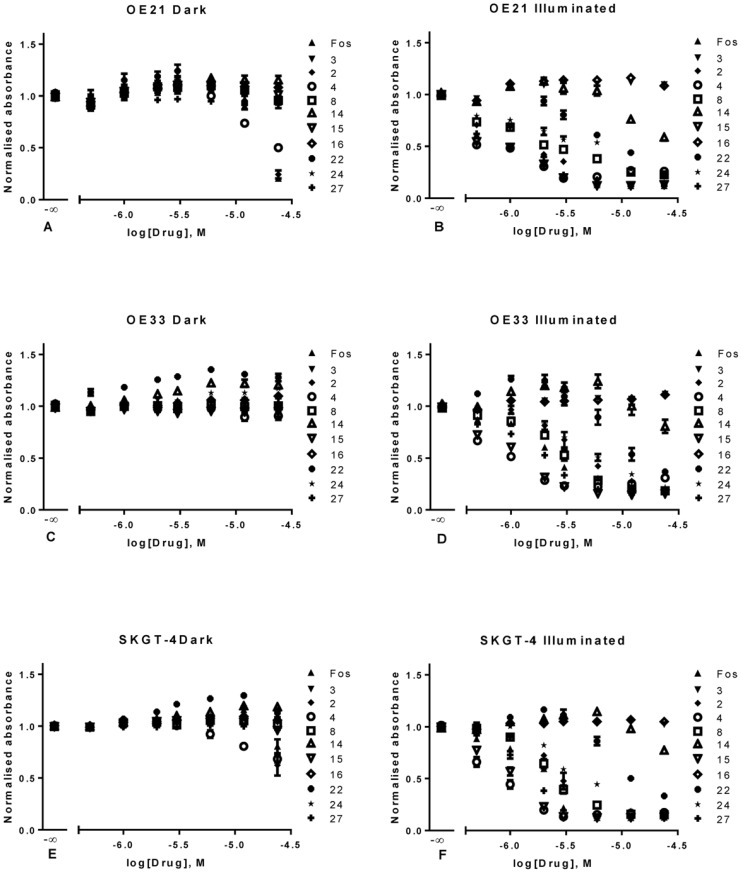
Graphical representation of the results obtained from the 24 hour MTS assay performed in the previously identified hits (compounds 2, 4, 8, 16, 22, 24 and 27) as well as three non-toxic compounds (3, 14 and 16) tested in OE21 cells (A and B) OE33 cells (C and D) and SKGT-4 cells (E and F). Data are representative of three independent experiments and values are expressed in mean ± SEM.

The identified hits (compounds from both screens) were then analyzed by MTS assay at a final post illumination time of 24 hours; this not only further validated our early timepoint of 4 hours but also allowed us to determine IC50 values for each of the hits identified in the previous screens. From these results were able to determine that the various cell lines do, in fact, respond to photodynamic treatments differently both in illuminated and dark conditions (Fig. 15). We were also able to ascertain that the results obtained at 4 hours are representative of a longer post illumination time as the IC50 values determined for OE21 cells remained unchanged (Table 2). The same could be inferred for the other cell lines. Of note is the fact that the chosen concentration range showed the beginning of dark toxicity at the higher 24 µM, allowing us to further compare the compounds analyzed.

**Table 2:** IC50 values determined for the various compounds screened and cells lines tested when illuminated.

	Foscan	3	2	4	8	14	15	16	22	24	27
**OE21**											
IC50 (µM)	2	5	2	2	2	1	2	7	7	3	2
r^2^	0.9	0.3	0.9	0.8	0.9	0.4	0.9	0.5	0.9	0.9	0.9
**OE33**											
IC50 (µM)	2	Interrupted	4	1	3	1	2	Interrupted	8	5	2
r^2^	0.9		0.9	0.9	0.9	0.4	0.9		0.8	0.9	0.9
**SKGT-4**											
IC50 (µM)	2	Interrupted	3	1	2	Ambiguous	1	Interrupted	8	4	2
r^2^	0.9		0.9	0.91	0.9		0.9		0.9	0.9	0.9

These were generated by fitting a non-linear regression (log(inhibitor) *versus* response with variable slope in Graphpad prism. Results are representative of three independent experiments and values were rounded to units.

From the results it can be seen that at this concentration OE21 cells are more susceptible to dark toxicity from Temoporfin and compound **2** whereas compound **4** showed less response than the positive control (Figure 15 A). No such dark toxicity was seen for OE33 cells making them much more resistant to these types of compounds (Fig. 15 C). The SKGT-4 cell line showed strong dark toxicity for Temoporfin, **2** and **4** with no significant difference between any of the three compounds (Fig. 15 E).

In addition, the results confirm the lack of toxicity of compounds **3**, **14** and **16**; if an IC50 could be determined the r^2^ values are too low in order for these to be deemed statistically valid (Table 2). These were considered to be an example of compounds that tested non-toxic in the primary screen.

In summary, the results herein presented allowed the development and validation of an improved methodology for drug primary screens. The information obtained using this methodology allows the evaluation of several biologic parameters, such as nuclear and cellular area. Furthermore, the time needed to evaluate the effects of the innumerous chemical compounds developed is greatly reduced. This new methodology will now be applied to a larger library of compounds.

## Conclusions

In this work we were committed to the development of a quick, reliable and multi-output method for performing primary screens on large libraries of novel compounds. Simple morphological parameters that have been extensively used by toxicologists for several years were chosen deliberately [5], [11], [48], [49]. Basic area measurements such as cell area, nuclear area and cell roundness can only change in three different ways: (i) decrease, (ii) increase, (iii) remain unchanged. The first two parameters can easily be identified using the proposed methodology and have been demonstrated to be good markers of bio-reactivity [48], [49]. If there is no change then any cytotoxicity will be detected later in the assay by assessing any changes in cell number.

The results illustrate the efficacy of evaluating potential photosensitizing agents quantitatively using high content imaging and analysis platform technologies. This lowers the number of required manipulations steps compared to other drugs and tests; it also reduces the overall time required for analysis and evaluation hence reducing experimental turnaround times. In addition, it is more cost effective than traditional testing as technological developments have made these types of equipment ever more available and inexpensive. Also, fixation allows for the storage of plates for subsequent staining (*e.g.*, organelle staining in order to clarify intracellular drug location) and for further multiplexing. Storage of images allows for re-interrogation in the future, should other questions arise. It is also important to note that these studies now allow the development of an automated high throughput screening protocol for the evaluation, QSAR studies, and cell biological investigation of photosensitizers, which is currently ongoing.

Phenotypic screens, based solely in the change of physical characteristics of cells, have recently been identified as the most effective method for failing drugs early in the research and development pipeline [48], [50]. This prevents significant financial expenditures on *in vivo* screens that would ultimately fail or, in a worst case scenario, if harmful, could lead to a total recall and withdrawal of the drug from the market. In fact, 37% of all “first-in-class” drugs discovered from 1999 to 2008 were discovered through phenotypic screens [51]. PDT development is an area mostly driven by individual research groups and not larger pharmaceutical companies with libraries. Most groups focus on small libraries of chemically closely related systems, mostly based on local experience or availability of dyes, often only with minor differences in structure from group to group. The use of HCA for large scale library screening of PS might offer new avenues for the identification of conceptionally different PS. HCA approaches as described here can offer significant advantages for the development of new PS drugs and other sensors and imaging compounds in photomedicine in general.
